# iTraNet: a web-based platform for integrated trans-omics network visualization and analysis

**DOI:** 10.1093/bioadv/vbae141

**Published:** 2024-09-30

**Authors:** Hikaru Sugimoto, Keigo Morita, Dongzi Li, Yunfan Bai, Matthias Mattanovich, Shinya Kuroda

**Affiliations:** Department of Biochemistry and Molecular Biology, Graduate School of Medicine, The University of Tokyo, Tokyo 113-0033, Japan; Department of Biological Sciences, Graduate School of Science, The University of Tokyo, Tokyo 113-0033, Japan; Molecular Genetics Research Laboratory, Graduate School of Science, The University of Tokyo, Tokyo 113-0033, Japan; Department of Biological Sciences, Graduate School of Science, The University of Tokyo, Tokyo 113-0033, Japan; Department of Biological Sciences, Graduate School of Science, The University of Tokyo, Tokyo 113-0033, Japan; Novo Nordisk Foundation Center for Basic Metabolic Research, University of Copenhagen, Copenhagen DK-2200, Denmark; Novo Nordisk Foundation Center for Biosustainability, Technical University of Denmark, Lyngby 2800, Denmark; Department of Biochemistry and Molecular Biology, Graduate School of Medicine, The University of Tokyo, Tokyo 113-0033, Japan; Department of Biological Sciences, Graduate School of Science, The University of Tokyo, Tokyo 113-0033, Japan

## Abstract

**Motivation:**

Visualization and analysis of biological networks play crucial roles in understanding living systems. Biological networks include diverse types, from gene regulatory networks and protein–protein interactions to metabolic networks. Metabolic networks include substrates, products, and enzymes, which are regulated by allosteric mechanisms and gene expression. However, the analysis of these diverse omics types is challenging due to the diversity of databases and the complexity of network analysis.

**Results:**

We developed iTraNet, a web application that visualizes and analyses trans-omics networks involving four types of networks: gene regulatory networks, protein–protein interactions, metabolic networks, and metabolite exchange networks. Using iTraNet, we found that in wild-type mice, hub molecules within the network tended to respond to glucose administration, whereas in *ob/ob* mice, this tendency disappeared. With its ability to facilitate network analysis, we anticipate that iTraNet will help researchers gain insights into living systems.

**Availability and implementation:**

iTraNet is available at https://itranet.streamlit.app/.

## 1 Introduction

A major goal of biology is a comprehensive understanding of molecular interactions within biological systems (Barabási and Oltvai 2004). These interactions intricately regulate intracellular processes, leading to the emergence of biological network visualization and analysis as pivotal methodologies in biology (Barabási and Oltvai 2004, Gehlenborg *et al.* 2010, Yugi *et al.* 2016, Waller *et al.* 2020, Zhou *et al.* 2022). Network visualization and analysis allow researchers to explore the relationships and interactions among molecules, uncovering the regulatory mechanisms that influence cellular behavior (Alon 2007, Yugi *et al.* 2016, Zhou *et al.* 2022).

One example of how analysis of the properties of entire biological networks has led to a better understanding of living systems is discovery of the robustness of “scale-free networks” to errors (Albert *et al.* 2000). Many complex systems, including metabolic networks, exhibit remarkable resilience to errors, with local failures rarely leading to a loss of global information transmission capacity within the networks (Albert *et al.* 2000). This resilience is partly due to the scale-free topology, characterized by heterogeneous wiring (Albert *et al.* 2000). Network analysis also guides hypothesis formation and the development of targeted intervention strategies by enabling the identification of pivotal nodes, hubs, and modules within complex networks (Yu *et al.* 2007, Zotenko *et al.* 2008, Liekens *et al.* 2011, Cho *et al.* 2014).

Biological networks include many forms, ranging from gene regulatory networks and protein–protein interactions (PPIs) to metabolic networks. Metabolic networks encompass substrates and products in metabolic reactions, as well as the enzymes that direct these processes. These enzymes are subject to regulation *via* allosteric regulation and gene expression regulation. Gene expression is regulated by transcription factors (TFs) and microRNAs (miRNAs). In addition, intracellular processes interact with extracellular molecules *via* transporters. These rationales underscore the importance of applying multilayered trans-omics networks to understanding biological systems (Yugi *et al.* 2014, 2016, Kawata *et al.* 2018, Kokaji *et al.* 2020, 2022; Egami *et al.* 2021; Terakawa *et al.* 2022).

However, analysing various omics layers still remains a laborious and time-consuming task due to their derivation from heterogeneous databases and the necessity for complex network analysis. Although some web applications exist to visualize networks of multiple types of molecules and perform multi-omics data analysis (Kokoli *et al.* 2023, Ewald *et al.* 2024), there is still a lack of established methods to comprehensively visualize and analyse trans-omics networks. In particular, no other web application integrates allosteric regulation and metabolite exchange between intracellular and extracellular spaces *via* transporters in a knowledge-driven manner, although these regulations play critical roles in metabolism. Ensuring the reproducibility of such studies is also challenging due to the continuously updating databases and occasional unavailability of publicly accessible codes for performing trans-omics analysis.

Addressing these challenges, we have developed iTraNet, a user-friendly and open-access web application for trans-omics network visualization and analysis. iTraNet is designed to visualize and analyse four key types of interactions in living systems: gene regulatory networks (including TF, miRNA, and mRNA), PPIs, metabolic networks (including enzyme, mRNA, and metabolite), and metabolite exchange networks (including transporter, mRNA, and metabolite). This application not only provides a visual representation of the intricate interplay among these molecules but also enables researchers to conduct analyses of their network properties, thereby offering a comprehensive understanding of the intricate biological dynamics.

## 2 Methods

### 2.1 Workflow overview

**Figure 1.**
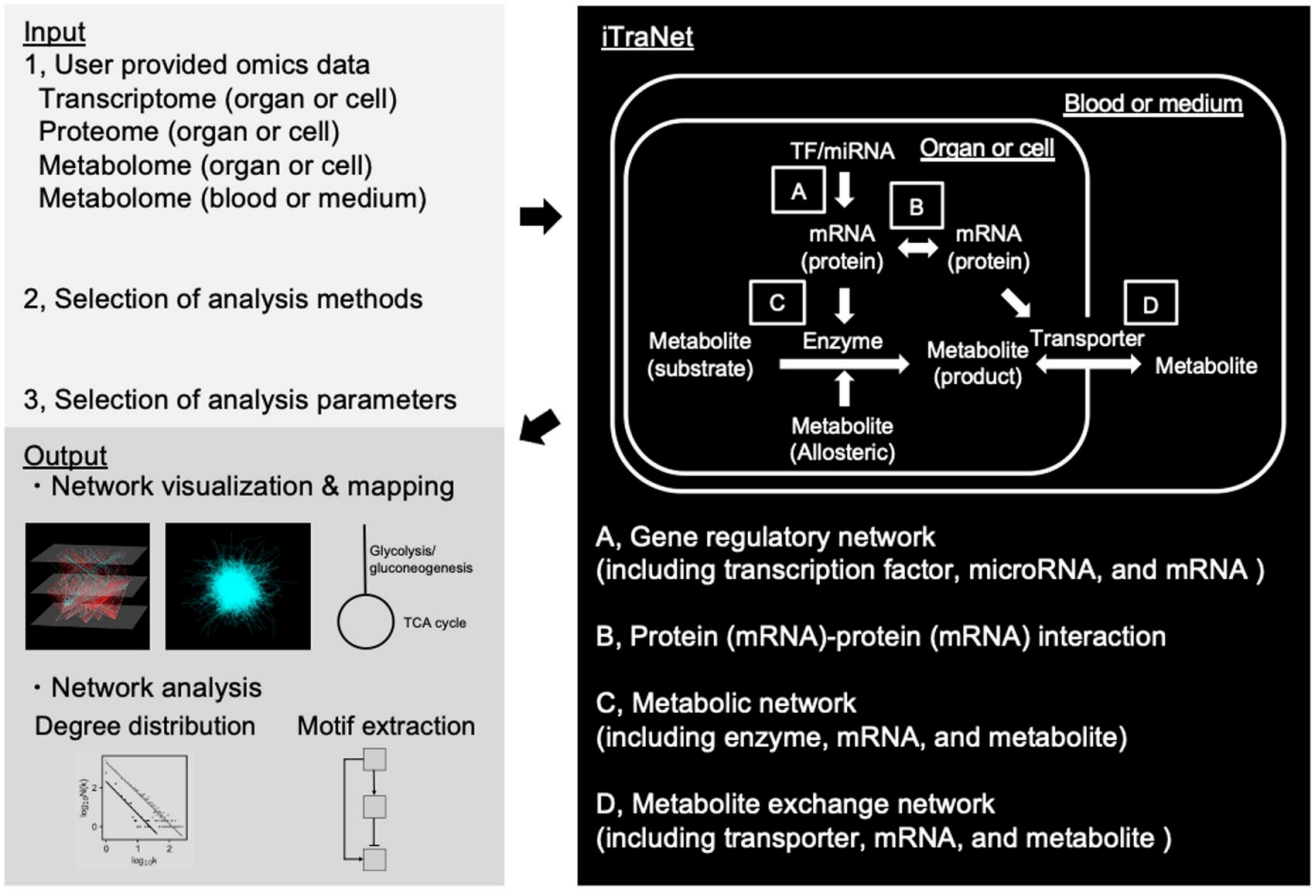
Schematic overview of iTraNet workflow. iTraNet requires transcriptome, proteome, and/or metabolome data, as input (left, top panel). iTraNet is designed to visualize and analyse four distinct categories of biological networks, as shown in the right panel: (A) gene regulatory networks [including transcription factors (TF), microRNA (miRNA), and mRNA); (B) protein (mRNA)–protein (mRNA) interactions; (C) metabolic networks (including enzyme, mRNA, and metabolite]; and (D) metabolite exchange networks (including transporter, mRNA, and metabolite). Finally, iTraNet outputs a comprehensive visualization of the four types of biological networks, maps for each metabolic pathway, and results of the network analyses (left, bottom panel).

The main aim of iTraNet is to provide researchers with an easily accessible platform for trans-omics network visualization and analysis. Figure 1 shows the overall workflow of iTraNet. To perform trans-omics network visualization and analysis in iTraNet, users are required to upload their own transcriptome, proteome, and/or metabolome data (Fig. 1, left panel). Upon submission, iTraNet performs visualization and analysis across four distinct categories of biological networks: (A) gene regulatory networks [including transcription factor (TF), microRNA (miRNA), and mRNA]; (B) protein (mRNA)–protein (mRNA) interactions; (C) metabolic networks (including enzyme, mRNA, and metabolite); and (D) metabolite exchange networks (including transporter, mRNA, and metabolite).

Estimating relationships within TF, miRNA, and mRNA networks (Fig. 1A) requires relevant transcriptome data from targeted organs or cells. Similarly, estimating relationships among mRNAs or proteins (Fig. 1B) necessitates transcriptome or proteome data from the targeted organs or cells. Meanwhile, assessing relationships involving enzymes, mRNAs, and metabolites (Fig. 1C) calls for both metabolome and transcriptome data from the targeted organs or cells. Estimating relationships among transporter, mRNA, and metabolite (Fig. 1D) requires metabolome and transcriptome data from the targeted organs or cells, as well as the associated blood or medium.

The following sections briefly summarize the approach used to construct and analyse trans-omics networks and its implementation as a web application. Supplementary texts 1 and 2 provide more details on the input and output of iTraNet. Comprehensive details on the construction and analysis of trans-omics networks can be found in our previous studies (Yugi *et al.* 2014, 2016, Kawata *et al.* 2018, Kokaji *et al.* 2020, 2022, Egami *et al.* 2021, Terakawa *et al.* 2022).

### 2.2 Gene regulatory network construction

**Figure 2.**
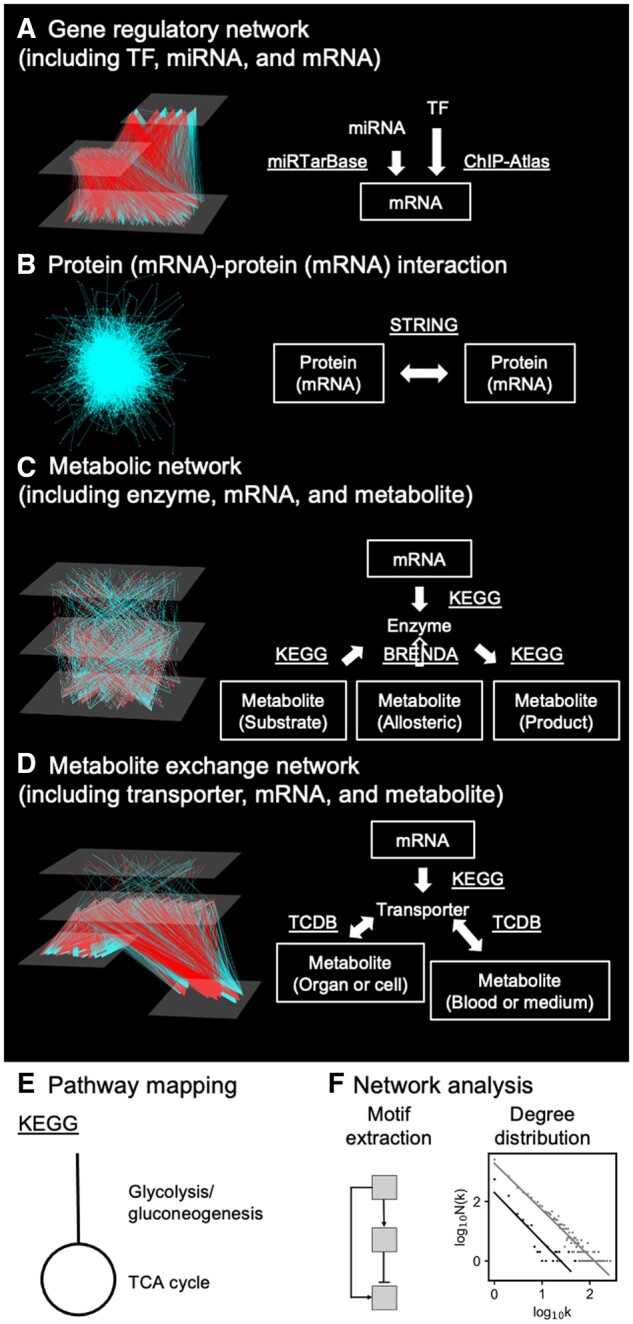
Outputs of iTraNet. (A) A network from transcription factor (TF) or microRNA (miRNA) to mRNA. TFs and miRNAs are estimated using the ChIP-Atlas and miRTarBase databases, respectively. (B) A network connecting mRNAs or proteins. The association is based on protein–protein interactions and is estimated by STRING. mRNAs can be used as proxies for proteins. (C) A network connecting mRNA, enzyme, and metabolite. The connections are estimated using KEGG and BRENDA. (D) A network connecting mRNA, transporter, and metabolite. The connections are estimated using KEGG and TCDB. (E) iTraNet visualizes trans-omics networks by arranging each metabolic pathway using the well-known KEGG layout. (F) iTraNet performs motif extraction from trans-omics networks and analyses topological features of trans-omics networks, such as degree distributions. The parameter $N\left(k\right)$ is the number of nodes in the network with k connections to other nodes.

iTraNet constructs gene regulatory networks by estimating TFs and miRNAs associated with the uploaded mRNAs (Fig. 2A). To estimate TFs, the ChIP-Atlas database (Zou *et al.* 2022) was employed, which includes publicly accessible ChIP-seq experiments. For each ChIP-seq experiment, the “Target Genes” information for Mus musculus (mm10) was extracted, identifying genes whose transcription start sites (TSSs) are bound to bait proteins (TFs). The distance at which binding occurs was set at 1 kb from the TSS. The threshold for statistical significance, calculated by the peak-caller MACS2, can be set by users. The enrichment of increased or decreased mRNAs in each ChIP-seq experiment was determined using a one-tailed Fisher’s exact test, followed by false discovery rate (FDR) correction using the Benjamini–Hochberg method. Users can set a threshold of FDR.

To estimate miRNAs associated with the uploaded mRNAs, miRTarBase (Huang *et al.* 2021) was employed. Mus musculus data in the miRTarBase was used for estimating miRNA.

### 2.3 Protein–protein interaction network construction

iTraNet estimates mRNAs or proteins associated with the uploaded mRNAs or proteins (Fig. 2B). The association is based on PPIs and is estimated using the STRING database (Szklarczyk *et al.* 2022), which provides a comprehensive resource for known and predicted PPIs. Only Mus musculus data from the STRING database was employed in iTraNet. The associations were determined based on a confidence score (Szklarczyk *et al.* 2022) derived from various types of evidence. iTraNet uses the score of 400, which is the default value in STRING.

### 2.4 Metabolic network construction

iTraNet estimates metabolic reactions and enzymes associated with the uploaded mRNAs and metabolites (Fig. 2C). Metabolic reactions are assumed to be catalysed by metabolic enzymes and affected by metabolites that function as the substrates, products, or allosteric regulators. Regulation of metabolic reactions also consists of regulations by changing the amount of enzyme through gene expression. This inference draws from analysis of data from Kyoto Encyclopedia of Genes and Genomes (KEGG) (Kanehisa *et al.* 2017) and BRaunschweig ENzyme DAtabase (BRENDA) (Chang *et al.* 2021). We extracted the allosteric regulatory network from the BRENDA database (https://www.brenda-enzymes.org/download.php). Activating and inhibiting compounds for each metabolic enzyme (reaction) in mammals were extracted and compound names were converted to KEGG ID using InChI key, KEGG compound name, or HMDB (https://hmdb.ca/downloads, ver. 5) (Wishart *et al.* 2022) compound name. These molecules are organized into a global metabolic pathway (mmu01100) in the KEGG database. Mus musculus data in KEGG database were used in iTraNet.

If users only have proteome data and not transcriptome data, the proteome data can be converted from ENSMUSP to ENSMUSG using Ensembl BioMarts (Kinsella *et al.* 2011), etc., and uploaded as transcriptome data for this analysis.

### 2.5 Metabolite exchange network construction

iTraNet also estimates transporters associated with the uploaded mRNAs, metabolites in the target organ or cell, and metabolites in blood or medium data (Fig. 2D). Transporters are assumed to be affected by metabolites and regulations by changing the amount of transporter gene expression. Transporters and their substrates were identified using the Transporter Classification Database (TCDB) (Saier *et al.* 2021). For substrates, we selected compounds that are associated with fewer than 50 other compounds, based on information from ChEBI IDs (Hastings *et al.* 2016). For transporters, we selected transporters that localize on cellular membrane, based on information from the UniProt database (The UniProt Consortium 2023).

If users only have proteome data and not transcriptome data, the proteome data can also be converted from ENSMUSP to ENSMUSG and uploaded as transcriptome data for this analysis.

### 2.6 Network visualization

iTraNet visualizes trans-omics networks by arranging each metabolic pathway, such as glycolysis/gluconeogenesis and the tricarboxylic acid (TCA) cycle using the well-known KEGG layout, thereby enhancing their interpretability (Fig. 2E). Of note, all network types described above are presented as interactive networks, enabling users to zoom in/out and drag nodes and edges as needed. More details about iTraNet input and output can be found in Supplementary texts 1 and 2.

### 2.7 Network analysis

To understand living systems, it is necessary not only to visualize and investigate how each molecule interacts but also to study the overall network properties of how all molecules interact, which goes beyond reductionist methods (Jeong *et al.* 2000, Barabási and Oltvai 2004). Moreover, network analysis identifying pivotal nodes, hubs, and modules within complex networks have guided hypothesis formation and developed targeted intervention strategies (Yu *et al.* 2007, Zotenko *et al.* 2008, Liekens *et al.* 2011, Cho *et al.* 2014).

To achieve such a comprehensive understanding of living systems, iTraNet calculates various properties of the trans-omics networks (Fig. 2F). In this web application, trans-omics networks are depicted as graphs where nodes represent various molecules, including mRNAs, miRNAs, proteins, or metabolites. Edges symbolize physical or functional interactions, following the typical representation (Atiia *et al.* 2020, Kokaji *et al.* 2020).

Given that a key characteristic of biological networks has been indicated as the majority-leaves minority-hubs (mLmH) topology (Barabási and Oltvai 2004, Atiia *et al.* 2020), iTraNet investigates the topological features of trans-omics networks. Of note, mLmH-based networks are often referred to as scale-free networks (Atiia *et al.* 2020); however, here, we employ the term mLmH to emphasize that this application neither assumes nor advocates the strict power-law distribution concept associated with scale-free networks, as it has generated some debate in the field (Atiia *et al.* 2020).

iTraNet calculates the degree and degree centrality of each node, as well as the degree distributions of trans-omics networks. The degree (*k*) of a node is defined as the number of connections or edges it has within the network, indicating its level of connectivity. “Degree centrality” (*C*_*D*_) quantifies the importance of a node based on the number of its connections, thereby providing insights into the node’s influence within the network. “Degree distributions” denote the distribution of nodes based on their degrees, indicating the pattern of connectivity across the network. iTraNet also calculates a scaling parameter (γ), which is defined as follows:

(1) Nk∝k-γ, 

where $N\left(k\right)$ denotes the number of nodes in the network with k connections to other nodes, and γ is estimated through least-squares fitting. Of note, commonly used methods for analysing power-law distributions, such as least-squares fitting, can yield imprecise γ (Clauset *et al.* 2009), and the value provided by iTraNet should be interpreted cautiously.

iTraNet also calculates several network metrics, including density, clustering coefficient, assortativity, and the mean degree of trans-omics networks. “Density (D)” refers to the ratio of the number of connected edges (E) present in a network to the total number of possible edges. This metric provides an indication of the extent to which the nodes within a network are interconnected. It is defined as follows:

(2) D=2ENN−1, 

where N denotes the number of nodes in the network. “Clustering coefficient” quantifies the extent to which nodes in a network tend to form clusters or groups. It measures the probability that the neighbors of a node are also connected to each other. Clustering coefficient of a node u (*C*_*u*_) is defined as follows:

(3) Cu=2T(u)D(u)(D(u)-1), 

where T(u) and D(u) represent the number of triangles through node u and the degree of u, respectively. “Assortative” refers to a network characteristic that describes the tendency of nodes with similar degrees to be connected. Positive assortative indicates that nodes with high degrees tend to connect with other nodes with high degrees, and nodes with low degrees connect to nodes with low degrees. “Mean degree” represents the average number of edges connected to nodes in a network. It provides an overall view of the network’s connectivity. These measures collectively indicate the structural properties and connectivity patterns within the trans-omics networks.

Biological networks can lead to nonlinearity in the input–output relationship when loops are formed, as exemplified by positive feedback (Angeli *et al.* 2004) and incoherent feedforward loop (Goentoro *et al.* 2009). Such network motifs can be associated with intriguing biological phenomena. Hence, iTraNet also incorporates the capability to extract loop structures from trans-omics networks.

### 2.8 Server implementation and code availability

The iTraNet web-based server is implemented in Python and uses the Streamlit library (https://www.streamlit.io) for the web application. iTraNet is hosted on the Streamlit cloud. The stand-alone tool to run iTraNet on a local machine is also available at the GitHub repository (https://github.com/HikaruSugimoto/Transomics_iTraNet). This code can be freely modified in a local machine to add or modify visualization and analysis methods.

iTraNet uses several Python libraries for data processing, network visualization, and analysis. Files are written using Pandas (Mc Kinney 2011). Trans-omics networks were visualized and analysed using Networkx (Hagberg *et al.* 2008), Matplotlib (Hunter 2007), and PyViz (an open-source visualization and analysis packages in Python https://pyviz.org/).

### 2.9 Case studies

To demonstrate the utility of iTraNet in visualizing and analysing trans-omics networks, two case studies were conducted using publicly available datasets. The first case study used publicly available transcriptome and metabolome data from mouse liver, muscle, and blood after the oral glucose tolerance test (Kokaji *et al.* 2020, 2022). These datasets included both wild-type (WT) and *ob/ob* mice that were fasted for 16 h before receiving glucose. The second case study examined publicly available transcriptome and metabolome data from brown adipose tissue (BAT) and blood samples from mice exposed to either room temperature (23°C) or cold (4°C) for 5 h (Panic *et al.* 2020).

## 3 Results

### 3.1 The iTraNet web server

iTraNet is an interactive web application developed using Streamlit (https://itranet.streamlit.app/). The application is also available for local use by using the code provided in the GitHub repository (https://github.com/HikaruSugimoto/Transomics_iTraNet). iTraNet is freely available to all users, and there is no login requirement. Users can directly upload their own data in a simple format that contains molecular IDs and types of responses (Supplementary texts 1 and 2).

iTraNet accepts transcriptome, proteome, and/or metabolome data as input. Users can also upload background genes for TF enrichment analysis. The output generated by iTraNet encompass four distinct types of network visualizations and analyses, as described in Section 2: (A) gene regulatory networks (including TF, miRNA, and mRNA) estimate TFs and miRNAs associated with the uploaded transcriptome data; (B) protein (mRNA)–protein (mRNA) interactions estimate proteins or mRNAs associated with the uploaded proteome or transcriptome data; (C) metabolic networks (including enzyme, mRNA, and metabolite) estimate metabolic reactions and enzymes associated with the uploaded transcriptome and metabolome data; and (D) metabolite exchange networks (including transporter, mRNA, and metabolite) estimate transporters associated with the uploaded transcriptome and metabolome data.

### 3.2 Case study 1: comprehensive analysis of the metabolic status after glucose administration

**Figure 3.**
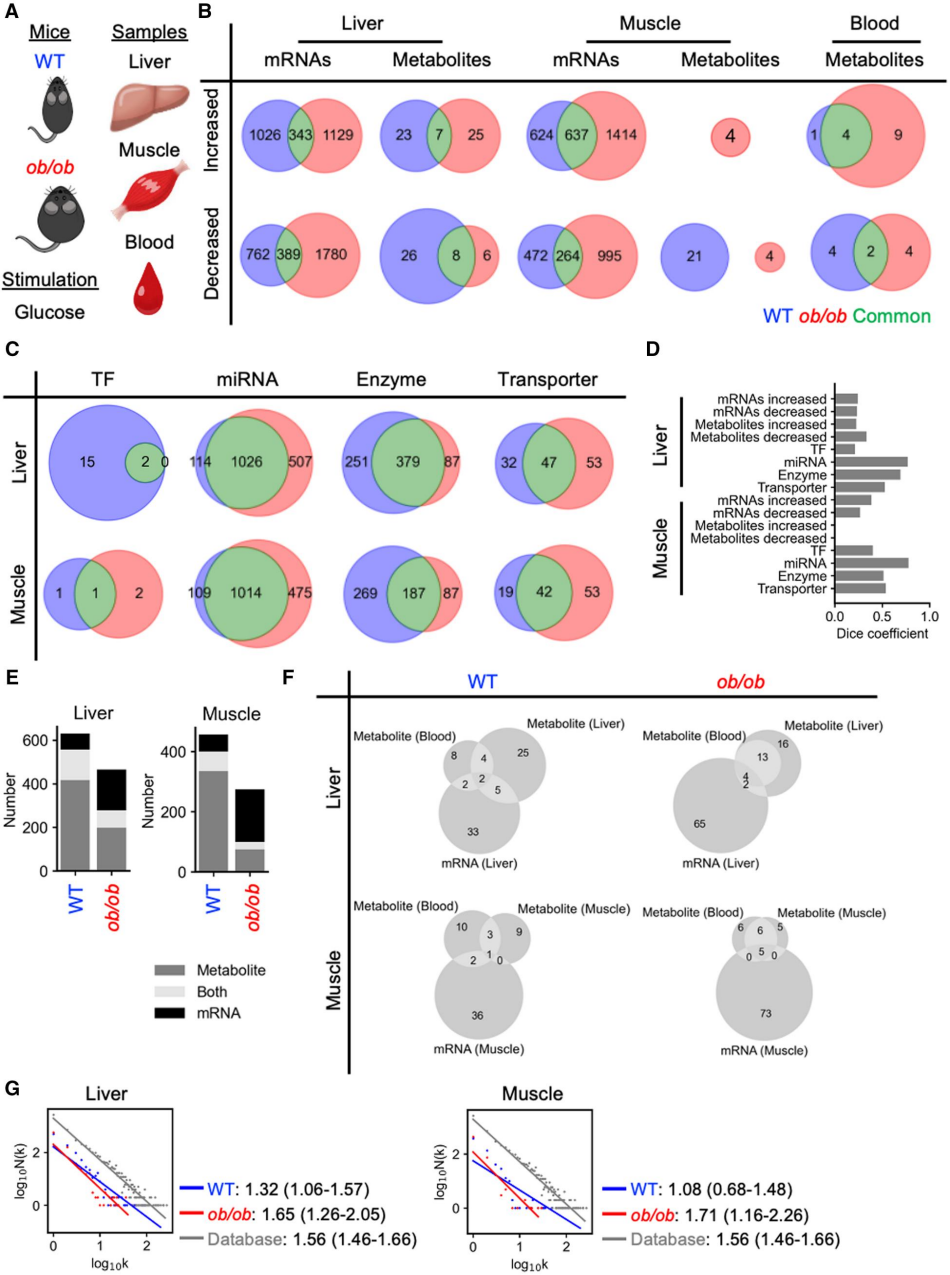
Comprehensive analysis of the metabolic status after glucose administration using iTraNet. (A) As a case study of iTraNet, we used previously reported transcriptome and metabolome data from WT and *ob/ob* mice liver, muscle, and blood after an oral glucose tolerance test. (B) The number of differentially expressed molecules in each condition. Molecules that exhibited changes specific to WT mice, changes specific to *ob/ob* mice, and changes in common to both WT and *ob/ob*. (C) The number of differentially expressed transcription factors (TFs), miRNAs, enzymes, and transporters predicted by iTraNet in each condition. (D) The dice coefficient of each Venn diagram shown in (B) and (C). A high value of the dice coefficient indicates the corresponding molecules are similar between the groups. (E) The number of predicted enzymes associated with the mRNAs, metabolites, or both in liver and muscle. In this analysis, metabolic reactions are assumed to be catalysed by metabolic enzymes and affected by metabolites that function as the substrates, products, or allosteric regulators. Regulation of metabolic reactions also consists of regulation by changing the amount of enzyme through gene expression. This inference draws from analysis of data from Kyoto Encyclopedia of Genes and Genomes (KEGG) and BRaunschweig ENzyme DAtabase (BRENDA). (F) The number of predicted transporters associated with the mRNAs, metabolites, or both in liver and muscle. Transporters are assumed to be affected by metabolites in the organs and in the blood, and regulations by changing the amount of transporter gene expression. This inference draws from analysis of data from the Transporter Classification Database (TCDB). (G) Degree distributions with fitted regression lines for the trans-omics network. *N*(*k*) represents the number of nodes in the network with k connections to other nodes. Gray, the degree distribution for the network consisting of all regulations in the KEGG and BRENDA databases; blue, the degree distribution for the network consisting of only differentially expressed reactions in WT; red, the degree distribution for the network consisting of only differentially expressed reactions in *ob/ob*. The values are the scaling parameters of the degree distributions, and the values in the parenthesis are 95% confidence interval.

To showcase the utility of iTraNet in visualizing and analysing trans-omics networks, we employed publicly available transcriptome and metabolome data from the mouse liver, muscle, and blood following the oral glucose tolerance test (Kokaji *et al.* 2020, 2022). In these datasets, 16-h fasted wild-type (WT) and *ob/ob* mice were orally administered glucose. Livers, muscles, and blood at 0, 20, 60, 120, 240 min after glucose administration were collected (Fig. 3A). The mRNAs and metabolites that exhibited significant changes after glucose administration, as determined by statistical methods described in previous studies, were used as input for iTraNet. The number of differentially expressed molecules is depicted in Fig. 3B.

Upon input submission, iTraNet estimated TFs, miRNAs, enzymes, and transporters associated with the differentially expressed molecules (Fig. 3C). To investigate which molecule types exhibited significant differences between WT and *ob/ob*, we calculated the dice coefficient, which increases if the corresponding molecules are similar between the groups (Fig. 3D). While the dice coefficient for mRNAs in the liver and muscle were comparable, the coefficient for liver metabolites was higher than that for muscle metabolites, suggesting distinct regulation of muscle metabolites in *ob/ob*. In both the liver and muscle, the dice coefficients for enzymes and transporters were larger than those for mRNAs and metabolites. These results indicate that in *ob/ob*, the response of mRNAs and metabolites to glucose was relatively different from that in WT; however, these mRNAs and metabolites were not completely dissimilar; rather, specific molecules among multiple mRNAs and metabolites associated with particular enzymes or transporters exhibited differences between WT and *ob/ob*.

iTraNet further estimated the number of enzymes associated with the mRNAs, metabolites or both (Fig. 3E). In both the liver and muscle, more enzymes were influenced by mRNAs, while fewer enzymes were affected by metabolites in *ob/ob*, as previously described (Kokaji *et al.* 2020, 2022). iTraNet also estimated the number of transporters associated with the mRNAs or metabolites or both (Fig. 3F). In both the liver and muscle, more transporters are affected by mRNA in *ob/ob*. Collectively, these results indicate that *ob/ob* exhibited an increased number of differentially expressed mRNAs affecting metabolic enzymes and transporters.

iTraNet also visualized the networks by arranging each metabolic pathway using the KEGG layout. To showcase the differences between the WT and *ob/ob* networks, we focused on the TCA cycle (Supplementary Fig. S7). In WT mice, positive allosteric regulation was observed in various enzymes throughout the TCA cycle, including 2.3.3.8, 1.1.1.41, 1.8.1.4, 6.2.1.6, and 1.3.5.1. Conversely, in *ob/ob* mice, only the enzyme 2.3.3.8 was positively regulated by allosteric mechanisms. This indicates a significant alteration in the allosteric regulation of the TCA cycle in *ob/ob* mice.

iTraNet also investigated the topological features of the trans-omics network. Among the analyses, degree distributions of the networks including enzyme, mRNA, and metabolite are shown in Fig 3G. The trans-omics network encompassing all regulations within the KEGG and BRENDA databases (Fig. 3G, gray line) can be considered a majority-leaves minority-hubs (mLmH) network. In both liver and muscle, the scaling parameters of the degree distribution of the WT network were smaller than those of the background network, indicating that WT tends to use hub nodes after glucose administration. By contrast, the scaling parameters of the degree distributions of the *ob/ob* networks were larger than those of the WT networks. Of note, standardized major axis tests revealed statistically significant differences in the regression lines between WT and *ob/ob* in both liver and muscle networks (*P *<* *.05 for both tissues). Hub nodes present only in the WT liver and muscle networks included ATP and UDP, which have been reported to be involved in many reactions as substrates, products, and allosteric regulators (Kokaji *et al.* 2020, 2022). Collectively, these results indicate that the hub nodes in WT tend to be unresponsive to glucose administration in *ob/ob*.

### 3.3 Case study 2: comprehensive analysis of the metabolic status under a cold environment

**Figure 4.**
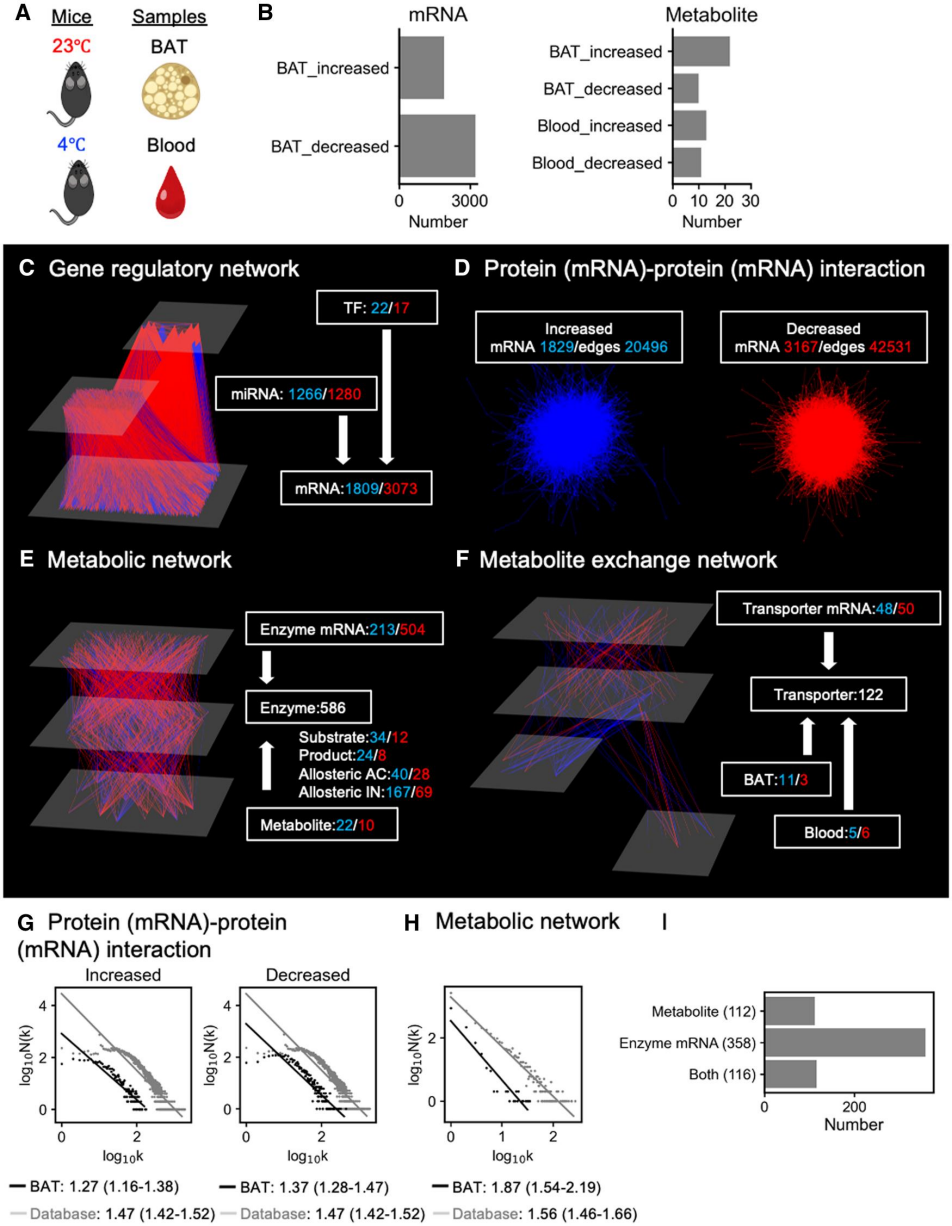
Comprehensive analysis of the metabolic status under the cold condition using iTraNet. (A) As a case study of iTraNet, we used previously reported transcriptome and metabolome data from brown adipose tissue (BAT) and blood from mice at room temperature (23°C) or cold (4°C). (B) The number of differentially expressed molecules in each condition. (C) A network connecting transcription factor (TF), microRNA (miRNA), and mRNA. TFs and miRNAs are estimated using iTraNet. Each number denotes the number of the associated molecules. Number with blue denotes the number of increased molecules under the cold condition, and that with red denotes the number of decreased molecules under the cold condition. (D) A network connecting mRNAs. The association is based on protein–protein interactions, and mRNAs are used as proxies for proteins. The blue network is created from mRNAs that increased in the cold environment, and the red one is created from mRNAs that decreased in the cold environment. (E) The trans-omics network for differentially regulated metabolic reactions. Nodes and edges indicate differentially expressed molecules and differential regulations, respectively. The Enzyme mRNA and Metabolite [Substrate, Product, Allosteric AC (activation), and Allosteric IN (inhibition)] layers correspond to differentially expressed mRNAs and differentially expressed metabolites, respectively. The Enzyme layer represents metabolic enzymes regulated by differentially expressed molecules. The differential regulations are classified into either activating (blue edges) or inhibiting (red edges). Increased Enzyme mRNA, increased Substrate, decreased Product, increased Allosteric AC, and decreased Allosteric IN are assumed to activate the reactions. (F) The trans-omics network for differentially regulated transporter. Nodes and edges indicate differentially expressed molecules and differential regulations, respectively. The Transporter mRNA and Metabolite (BAT and Blood) layers correspond to differentially expressed transcripts and differentially expressed metabolites, respectively. The Transporter layer represents transporters associated with differentially expressed molecules. The differential regulations are classified into either activating (edges with blue) or inhibiting (edges with red). Increased transporter mRNAs and increased metabolites are assumed to activate the reactions. (G) Degree distributions with fitted regression lines for the mRNA–mRNA network, which is based on protein–protein interactions shown in (D). *N*(*k*) represents the number of nodes in the network with *k* connections to other nodes. Gray, the degree distribution for the network consisting of all regulations in the STRING database; black, the degree distribution for the network consisting of only differentially expressed relation in BAT. The values are the scaling parameters of the degree distributions, and the values in the parenthesis are 95% confidence interval. (H) Degree distributions with fitted regression lines for the trans-omics network shown in (E). *N*(*k*) represents the number of nodes in the network with k connections to other nodes. Gray, the degree distribution for the network consisting of all regulations in the KEGG and BRENDA databases; black, the degree distribution for the network consisting of only differentially expressed reactions in BAT. The values are the scaling parameters of the degree distributions, and the values in the parenthesis are 95% confidence interval. (I) The number of predicted enzymes associated with the mRNAs or metabolites or both in BAT.

To further showcase the utility of iTraNet in visualizing and analysing trans-omics networks, we employed another publicly available transcriptome and metabolome data (Panic *et al.* 2020). In this dataset, brown adipose tissue (BAT) and blood from mice at room temperature (23°C) or cold (4°C) for 5 h were investigated (Fig. 4A). The mRNAs and metabolites were comprehensively examined by RNA sequencing (RNA-seq) and gas chromatography–mass spectrometry (MS), respectively. The mRNAs and metabolites that exhibited significant changes between the two groups, as determined by statistical methods described in the previous study, were used as input for iTraNet. The number of significantly increased and decreased molecules under the cold condition is depicted in Fig. 4B.

Upon input submission, iTraNet generated and visualized four types of biological networks (Fig. 4C–F). Table 1A shows the TFs and miRNAs associated with many mRNAs in the gene regulatory network (Fig. 4C). These included previously reported key relationships in BAT function and thermogenesis, including Pparg (TF) and *Ucp1* (mRNA) (Lasar *et al.* 2018). These included not only the intracellular relationship, but also the relationship between blood metabolites and BAT, such as increased blood glycerol levels and its transporter, *Aqp7*.

**Table 1.** List of molecules with high degrees.^a^

A		B		C		D	
TF/miRNA	Degree	Increased mRNA	Degree	Decreased mRNA	Degree	Metabolite	Degree
Kmt2d	4282	Jun	177	Trp53	444	Succinate	33
Brd4	4262	Hsp90ab1	169	Kras	322	Adenosine	31
Creb1	4069	Ubc	168	Hras	305	Asp	29
Pparg	4001	Dhx15	140	Cdc42	288	D-Glucose 6-phosphate	25
Crebbp	3742	Atxn1	137	Il6	286	Glycerol	21
Ep300	3560	Hspa5	136	Alb	258	Cholesterol	21
Hdac3	3412	Mapk1	135	Cd4	248	Leu	20
Ppara	3300	Eprs	127	Mapk14	225	Phe	19
Ncor1	3163	Eif4a3	127	Rac1	219	Ser	19
Cebpa	3143	Polr1a	126	Ptprc	217	2,3-Bisphosphoglycerate	18
Nr1d1	2956	Nop58	123	Gsk3b	214	Octadecanoic acid	17
Cebpb	2858	Pa2g4	119	Cdk1	208	Val	13
Prdm16	2720	Gtpbp4	118	Atm	207	Uridine	13
Ebf2	2631	Pno1	117	Il1b	201	Adenine	12
Tle3	2562	Hspa4	113	Rac2	200	Ile	8

a List of 15 molecules with high degrees in the networks of Fig. 4C (A), Fig. 4D, increased (B), Fig. 4D, decreased (C), and Fig. 4E (D), respectively.

iTraNet also estimated the protein (mRNA)–protein (mRNA) interactions (Fig. 4D) and investigated the topological features of the network (Fig. 4G). The protein (mRNA)–protein (mRNA) interaction network including all relationships within the STRING databases (Fig. 4G, gray line) can be considered a mLmH network with a power-law degree distribution. In both the increased and decreased networks, the scaling parameters of the degree distributions were smaller than those in the background network (Fig. 4G), indicating that mice tend to use hub mRNA nodes under the cold condition. By contrast, the scaling parameter of the enzyme, mRNA, and metabolite network was larger than that of the background network (Fig. 4H), indicating that mice did not tend to use the hub nodes of the enzymes, mRNAs, and metabolite network under the cold condition.

iTraNet also estimated the number of enzymes associated with the mRNAs, metabolites, or both (Fig. 4I). Although many metabolites including glucose and amino acid metabolism were significantly changed in their concentrations both after glucose loading (Kokaji *et al.* 2020, 2022) and cold stimulation (Panic *et al.* 2020), many enzymes were found to be affected by metabolite changes after glucose loading (Fig. 3E) and by mRNA changes under cold stimulation in WT mice (Fig. 4I). These results collectively indicate that the tendency to use hub nodes of biological networks and mRNAs or metabolites in regulating metabolic reactions can vary by organ or condition. For a deeper understanding of metabolism, we can also use iTraNet to look up the names of the nodes in order of increasing degree in each network (Table 1).

## 4 Discussion

iTraNet stands as a user-friendly interactive web application designed for visualizing and analysing trans-omics networks. This web application is capable of estimating TFs, miRNAs, enzymes, and transporters associated with transcriptome, proteome, and/or metabolome data uploaded by users. It also visualizes four distinct interactive biological networks: (A) gene regulatory networks (including TF, miRNA, and mRNA); (B) protein (mRNA)–protein (mRNA) interactions; (C) metabolic network (enzyme, mRNA, and metabolite); and (D) metabolite exchange networks (including transporter, mRNA, and metabolite). Additionally, the web application conducts analyses of network properties, exemplified by degree distributions as demonstrated in the case studies.

We used iTraNet to investigate the metabolic status after glucose loading (Fig. 3). In WT mice, hub molecules within the trans-omics network tended to respond to glucose administration, whereas this tendency disappeared in *ob/ob* mice. Metabolic networks have a mLmH topology, which is remarkably resilient to errors, with local errors rarely leading to a loss of global information transmission capacity within the network due to the presence of hub molecules (Albert *et al.* 2000). Therefore, disruption of homeostasis in biological networks with mLmH topology may inevitably involve changes in the configuration of the overall topology of the network, including changes in hub molecules, as observed in *ob/ob* mice. Of note, although the coverage of metabolites that can be measured by MS is high, it is less comprehensive than RNA-seq. Due to this coverage difference, the main focus in examining the effects of mRNAs and metabolites on enzymes will be on relative comparisons, such as the comparison between WT and *ob/ob* mice, as described in this study.

We also investigated the metabolic status under cold stimulation (Fig. 4) using iTraNet. Both glucose administration (Kokaji *et al.* 2020, 2022) and cold stimulation (Panic *et al.* 2020) affect various metabolites, including glucose and amino acid metabolism, but the regulation of these molecules and the overall topology of the networks might be different between the two conditions (Figs. 3 and 4). The tendency to use hub nodes in biological networks and the tendency to use either mRNAs or metabolites to regulate metabolic reactions varied among organs or conditions. Such a comprehensive understanding and overview of living systems requires comprehensive molecular measurements and trans-omics analysis, as facilitated by this web application. Network analyses have also identified pivotal nodes within complex networks and have guided hypothesis generation (Yu *et al.* 2007, Zotenko *et al.* 2008, Liekens *et al.* 2011, Cho *et al.* 2014). For this purpose, iTraNet can also output the name of each hub node, as shown in Table 1, which includes previously reported key molecules in BAT function. Collectively, we expect that iTraNet can facilitate researchers in understanding biological dynamics both from the perspective of the overall network properties and from the perspective of individual molecular interactions.

Various web applications have been developed for visualizing and analysing multi-omics data such as OmicsNet (Zhou and Xia 2018, Zhou *et al.* 2022, Ewald *et al.* 2024), 3Omics (Kuo *et al.* 2013), PaintOmics (Hernández-de-Diego *et al.* 2018), MergeOmics (Ding *et al.* 2021), MiBiOmics (Zoppi *et al.* 2021), OmicsAnalyst (Zhou *et al.* 2021), and Arena3D (Karatzas *et al.* 2021, Kokoli *et al.* 2023). The detailed characteristics of these web-based tools were well summarized and compared previously (Zhou *et al.* 2022, 2021). Compared to conventional web applications such as OmicsNet, one of iTraNet’s distinct strengths lies in its capacity to arrange multi-omics molecules within each metabolic pathway, such as glycolysis/gluconeogenesis and the TCA cycle, using the well-known KEGG layout. This feature is anticipated to facilitate a more intuitive understanding of intricate biological networks by researchers. Moreover, iTraNet can estimate more various regulations, such as allosteric regulation and regulation by transporters. More details on the comparison of iTraNet with other tools can be found in Supplementary text 3. We expect that this web application can deepen the understanding of biological phenomena by visualizing individual molecules on the well-known metabolic map and simultaneously analysing the overall features of the trans-omics networks that include more various regulations.

The current web server has several limitations. Due to storage constraints, iTraNet is currently designed exclusively for Mus musculus data. However, it is possible to apply the code in this web application to other species while maintaining the same framework, and the code in the GitHub repository (https://github.com/HikaruSugimoto/Transomics_iTraNet) can be downloaded and modified to analyse other species data. For species that are well characterized in the databases we use, the adaptation of iTraNet is relatively straightforward. In these cases, the primary task is to modify the existing Mus musculus-specific code to handle data corresponding to the target species, such as Homo sapiens. This approach allows us to maintain a high level of accuracy and reliability in the results. For species with less extensive data availability, we acknowledge that the process becomes more complex and may result in reduced accuracy. This involves converting genes of the target species to their corresponding Mus musculus orthologs, which can then be used as input to iTraNet. Of note, the KEGG database requires a license for nonacademic users (https://www.pathway.jp/en/licensing.html). Moreover, the web application is provided with limited memory, which might lead to slower access and computations. For faster and more stable calculations, users can download the code from the GitHub repository (https://github.com/HikaruSugimoto/Transomics_iTraNet) and run iTraNet on a local machine. iTraNet takes lists of mRNAs and metabolites as input, and the output of RNA-seq and MS cannot be directly used as the input; however, transcriptome and metabolome data can be analysed using other well designed web servers, such as iDEP (Ge *et al.* 2018) and MetaboAnalyst (Pang *et al.* 2022). Furthermore, this web application still estimates a limited kind of molecular interactions. Future development of a web application that includes other molecular interactions, such as DNA methylation, long non-coding RNA, and phosphorylation is warranted.

In conclusion, we have introduced iTraNet, a web application that provides users with visualizations and analyses of trans-omics networks. Given its ability to easily visualize the intricate interactions of the various biological molecules and perform trans-omics network analyses, we expect that it will facilitate researchers in understanding complex biological dynamics.

## Supplementary Material

vbae141_Supplementary_Data

## Data Availability

iTraNet is freely available for all users at https://itranet.streamlit.app/, and the source code is available at the GitHub repository (https://github.com/HikaruSugimoto/Transomics_iTraNet). The stand-alone tool to run iTraNet on a local machine is also available at the GitHub repository (https://github.com/HikaruSugimoto/Transomics_iTraNet).
