# Supplementary material for: iTraNet: a web-based platform for integrated trans-omics network visualization and analysis
Source: Bioinform Adv. 2024 Sep 30;4(1):vbae141. doi: 10.1093/bioadv/vbae141 (PMC11493990; doi:10.1093/bioadv/vbae141)
Supplement: vbae141_Supplementary_Data [file vbae141_supplementary_data.pdf]

Supplementary Texts

1. Inputs of iTraNet

This supplementary text outlines the input requirements for iTraNet, which include transcriptome, proteome and/or metabolome data in the specified format (Fig. S1). Demo data can be downloaded from iTraNet (https://itranet-a.streamlit.app/). Users are required to provide lists of mRNAs (ENSMUSG), proteins (ENSMUSP), and/or metabolites (CPD) for analysis of their relationships. As shown in the case studies in the main text, the primary goal of iTraNet is to analyze mRNAs, proteins, and metabolites measured by RNA-seq or mass spectrometry. However, iTraNet can also predict interactions with user-uploaded molecules. Importantly, direct upload of RNA-seq and mass spectrometry output is not supported by iTraNet, but such data can be processed using other tools such as iDEP (Ge *et al.*, 2018) and MetaboAnalyst (Pang *et al.*, 2022). ID conversion can also be performed using other tools such as Ensembl BioMarts (Kinsella *et al.*, 2011).

ENSMUSG	FC	ENSMUSP	FC	CPD	FC
ENSMUSG00000000028	Down	ENSMUSP00000000049	UP	C03793	Down
ENSMUSG00000000127	Down	ENSMUSP00000000090	UP	C05123	Down
ENSMUSG00000000247	Down	ENSMUSP00000000466	UP	C05145	Down
ENSMUSG00000000266	Down	ENSMUSP00000000608	UP	C05984	Down
ENSMUSG00000000708	Down	ENSMUSP00000001027	UP	C00002	UP
ENSMUSG00000000804	Down	ENSMUSP00000001183	UP	C00015	UP
ENSMUSG00000000838	Down	ENSMUSP00000001452	UP	C00044	UP
ENSMUSG00000001095	Down	ENSMUSP00000001479	UP	C00047	UP
ENSMUSG00000001166	Down	ENSMUSP00000001507	UP	C00048	UP

Figure S1. A list of mRNAs (ENSMUSG), proteins (ENSMUSP) and/or metabolites (CPD) that can be used as input to iTraNet.

Upon submission, iTraNet performs visualization and analysis across four different categories of biological networks: (A) gene regulatory networks (including transcription factor (TF), microRNA (miRNA), and mRNA); (B) protein (mRNA)-protein (mRNA) interactions; (C) metabolic networks (including enzyme, mRNA, and metabolite); and (D)

metabolite exchange networks (including transporter, mRNA, and metabolite), as shown in Figure 2. Users have the flexibility to select any of these categories in any order. Users can change the color of the networks in the “Set parameters” section (Fig. S2) and adjust parameters such as the false discovery rate for transcription factor estimation, as described in the Methods.

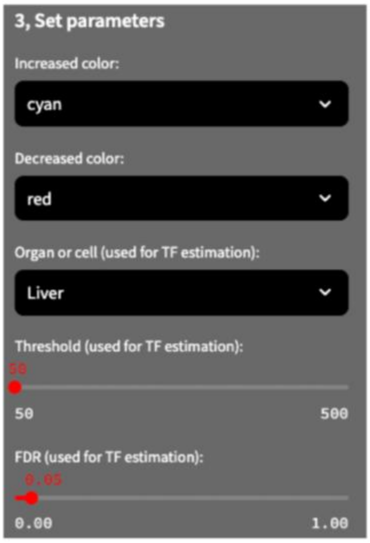

Figure S2. Users can change some parameters of the networks in “Set parameters”.

Of note, users have the option to download the code from the GitHub repository (https://github.com/HikaruSugimoto/Transomics_iTraNet) and run iTraNet on a local machine. The current version of iTraNet uses databases information available prior to October 2023. Users can update the database information files in the Database folder (https://github.com/HikaruSugimoto/Transomics_iTraNet) on the user’s local machine to ensure that the trans-omics networks are constructed using the most recent data available. This feature ensures that the generated networks accurately reflect the current state of knowledge in the field, as the databases are continually expanded with newly discovered interactions and annotations. Furthermore, when we update the versions of the databases used in iTraNet in the future, we will notify users through both the web application home page and the GitHub repository. In addition, we plan to maintain the older versions of the databases to ensure that users can access previous data if needed. For further details on the construction and analysis of trans-omics networks, comprehensive information can be found in our previous studies (Kokaji *et al.*, 2022, 2020; Egami *et al.*, 2021; Terakawa *et al.*, 2022; Yugi *et al.*, 2014; Kawata *et al.*, 2018; Yugi *et al.*, 2016).

2. Outputs of iTraNet

The output of iTraNet includes four types of network visualizations and analyses, as described in the main text: (A) gene regulatory networks, which estimate TFs and miRNAs associated with the uploaded transcriptome data; (B) protein (mRNA)–protein (mRNA) interactions, which estimate proteins or mRNAs associated with the uploaded proteome or transcriptome data; (C) metabolic networks, which estimate metabolic reactions and enzymes associated with the uploaded transcriptome and metabolome data; and (D) metabolite exchange networks, which estimate transporters associated with the uploaded transcriptome and metabolome data. We used the demo data from iTraNet in the following sections.

2A. Gene regulatory networks

When users select “(A) gene regulatory networks” in iTraNet, iTraNet estimates TFs and miRNAs associated with the uploaded mRNAs (Fig. S3). The associations are estimated using the ChIP-Atlas (Zou *et al.*, 2022) and miRTarBase (Huang *et al.*, 2021) databases, respectively. iTraNet generates a table of differentially expressed mRNAs along with associated TFs and miRNAs (Fig. S3A), which can be download as a CSV file. iTraNet also generates an interactive trans-omics network connecting the molecules, allowing users to

zoom in/out and drag nodes and edges as needed (Fig. S3B).

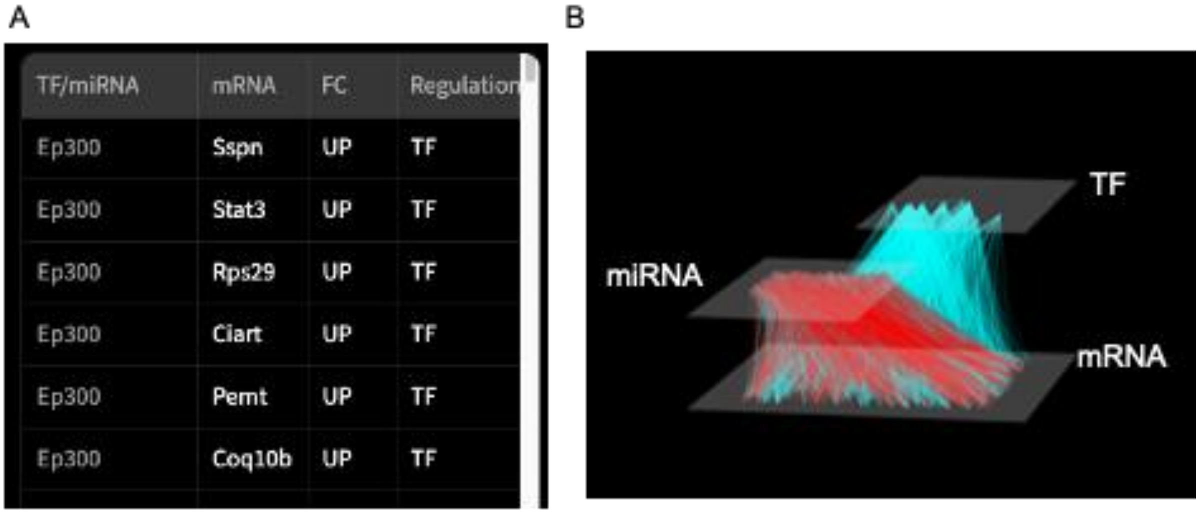

Figure S3. Outputs of "(A) gene regulatory network".
(A) A table of differentially expressed mRNAs and associated transcription factors (TFs) and miRNAs. The associated TFs and miRNAs are estimated using the ChIP-Atlas (https://chip-atlas.org/enrichment_analysis) and miRTarBase (https://mirtarbase.cuhk.edu.cn/~miRTarBase/miRTarBase_2022/php/download.php) databases, respectively.
(B) The trans-omics network for differentially expressed mRNAs and associated transcription factors (TFs) and miRNAs. Nodes and edges indicate differentially expressed molecules and differential regulations, respectively. iTraNet also outputs an interactive network that allows users to move molecules contained in TF, miRNA, and mRNA layers.

2B. Protein (mRNA)–protein (mRNA) interactions

When users select "(B) Protein (mRNA)–protein (mRNA) interactions" in iTraNet, iTraNet estimates proteins or mRNAs associated with the uploaded mRNAs or proteins (Fig. S4). The associations are based on PPIs and are estimated using the STRING database (Szklarczyk *et al.*, 2022). iTraNet generates tables of differentially expressed proteins (mRNAs) and associated proteins (mRNAs) (Fig. S4A) and tables of PPI network properties (Fig. S4B, C), which can be download as CSV files. iTraNet also generates interactive networks connecting the molecules, allowing users to zoom in/out and drag nodes and edges as needed (Fig. S4D).

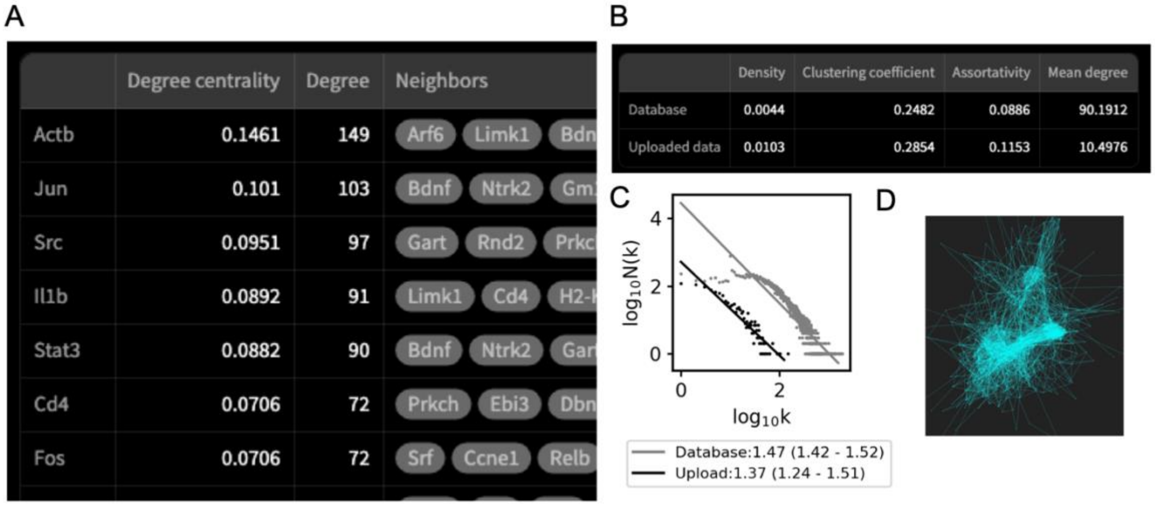

A

	Degree centrality	Degree	Neighbors
Actb	0.1461	149	Arf6 Limk1 Bdn
Jun	0.101	103	Bdnf Ntrk2 Gm
Src	0.0951	97	Gart Rnd2 Prkc
Il1b	0.0892	91	Limk1 Cd4 H2-
Stat3	0.0882	90	Bdnf Ntrk2 Gar
Cd4	0.0706	72	Prkch Ebi3 Dbn
Fos	0.0706	72	Srf Ccne1 Relb

B

	Density	Clustering coefficient	Assortativity	Mean degree
Database	0.0044	0.2482	0.0886	90.1912
Uploaded data	0.0103	0.2854	0.1153	10.4976

Figure S4. Outputs of ”(B) Protein (mRNA)–protein (mRNA) interactions”.
(A) A table of differentially expressed proteins (mRNAs) and associated proteins (mRNAs). The association is based on protein-protein interactions and is estimated using the STRING database (https://string-db.org/cgi/download?sessionId=b7GipzHI6zXz). Each protein (mRNA) is listed in descending order of degree in the protein (mRNA)–protein (mRNA) interactions (PPI) network. Users can sort them alphabetically by clicking on the first row.
(B) A table of the PPI network properties. Density, clustering coefficient, assortativity, and mean degree of the PPI network.
(C) Degree distributions with fitted regression lines for the PPI network. N(k) represents the number of nodes in the network with k connections to other nodes. The values are the scaling parameters of the degree distributions, and the values in the parenthesis are the 95% confidence intervals.
(D) The PPI network for differentially expressed proteins (mRNAs) and associated proteins (mRNAs). Nodes and edges indicate differentially expressed molecules and differential regulations, respectively. iTraNet also outputs the interactive network.

2C. Metabolic network

When users select “(C) Metabolic network” in iTraNet, iTraNet estimates metabolic reactions and enzymes associated with the uploaded mRNAs and metabolites (Fig. S5). Metabolic reactions are assumed to be catalyzed by metabolic enzymes and affected by metabolites that function as the substrates, products, or allosteric regulators. Regulation of metabolic reactions also consists of regulations by changing the amount of enzyme through gene expression. This inference is based on data analysis from Kyoto Encyclopedia of Genes and Genomes (KEGG) (Kanehisa *et al.*, 2017) and BRaunschweig ENzyme DAtabase (BRENDA) (Chang *et al.*, 2021).

iTraNet generates tables of differentially regulated metabolic reactions and associated differentially expressed molecules, as well as tables of metabolic network

properties (Fig. S5A, B), which can be download as CSV files. Biological networks can lead to nonlinearity in the input–output relationship when loops are formed, as exemplified by the incoherent feedforward loop (Goentoro *et al.*, 2009). Such network motifs can be associated with intriguing biological phenomena. To investigate network motifs, iTraNet extracts loop structures from the networks (Fig. S5C). iTraNet also outputs interactive networks connecting the molecules, allowing users to zoom in/out and drag nodes and edges as needed (Fig. S5D), and outputs the number of differentially regulated metabolic reactions regulated by metabolites, mRNAs, or both (Fig. S5E). In addition, iTraNet allows visualization of the degree distributions of the networks (Fig. S5F), and visualizes trans-omics networks by arranging each metabolic pathway, such as glycolysis/gluconeogenesis and the tricarboxylic acid (TCA) cycle using the well-known KEGG layout, thereby enhancing their interpretability (Fig. S5G).

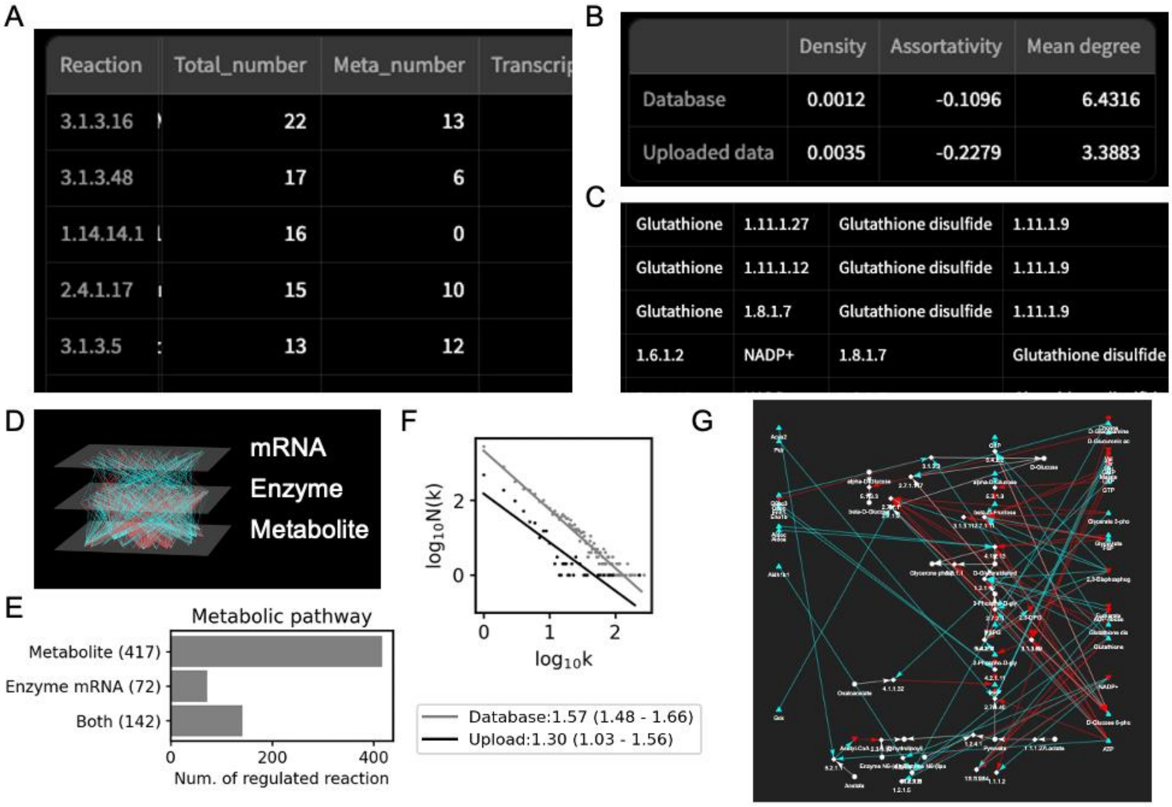

Figure S5. Outputs of ”(C) Metabolic networks”.
(A) A table of differentially regulated metabolic reactions and associated differentially expressed molecules. Metabolic reactions are assumed to be catalyzed by metabolic enzymes and affected by metabolites that function as the substrates, products, or allosteric regulators. Regulation of metabolic reactions also consists of regulations by changing the amount of enzyme through gene expression. This inference draws from analysis of data from KEGG API (https://www.kegg.jp/kegg/rest/) and BRENDA (https://www.brenda-enzymes.org/oldstart.php). Each reaction is listed in descending order of degree in the metabolic network. “Total_number” refers to the total number of connections between a given reaction and the associated molecules that show differential expression patterns. On the other hand, ”Meta_number” specifically represents the total number of connections between a reaction and its associated differentially expressed metabolites.
(B) A table of the metabolic network properties. Density, assortativity, and mean degree of the metabolic network.
(C) List of nodes forming loops in the metabolic network consisting of only differentially regulated reactions. Each row lists a set of molecules that collectively form a loop within the trans-omics network. For example, the first row shows a loop consisting of four molecules: glutathione, 1.11.1.27, glutathione disulfide, and 1.11.1.9. This loop represents a cyclic interaction pathway in which glutathione is linked to reaction 1.11.1.27, which is then linked to glutathione disulfide. The loop is completed by coupling of glutathione disulfide to reaction 1.11.1.9, which in turn is coupled back to glutathione.
(D) The metabolic network for differentially regulated metabolic reactions. Nodes and edges indicate differentially expressed molecules and differential regulated reactions, respectively. iTraNet also displays the interactive network that allows users to move molecules contained in mRNA, Enzyme, and Metabolite layers.
(E) The number of differentially regulated metabolic reactions that are regulated by metabolites, enzyme mRNAs, or both in the metabolic network.
(F) Degree distributions with fitted regression lines for the metabolic network. N(k) represents the number of nodes in the network with k connections to other nodes.
(G) The metabolic networks for differentially regulated metabolic reactions in each pathway (glycolysis/gluconeogenesis in this figure). Nodes and edges indicate differentially expressed molecules and differentially regulated reactions, respectively. The differential regulations are classified into either activating (edges with “increased color” (cyan)) or inhibiting (edges with “decreased color” (red)). Arrows from metabolites in the right column to metabolic enzymes represent allosteric regulation, while arrows from mRNAs in the left column to metabolic enzymes indicate regulation by changes in mRNA levels.

2D. Metabolite exchange network

When users select “(D) Metabolite exchange network” in iTraNet, iTraNet estimates transporters associated with the uploaded mRNAs, metabolites in the target organ or cell, and metabolites in blood or medium (Fig. S6). Transporters are assumed to be affected by metabolites and regulations by changing the amount of transporter gene expression. This inference draws from analysis of data from the Transporter Classification Database (TCDB) (Saier *et al.*, 2021). iTraNet outputs tables of differentially regulated transporters and associated differentially expressed molecules (Fig. S6A), which users can download as CSV

files. iTraNet also outputs interactive networks connecting the molecules, allowing users to zoom in/out and drag nodes and edges as needed (Fig. S6B), and outputs the number of differentially regulated transporters that are regulated by the metabolites in blood or medium, the metabolites in the organ or cells, mRNAs, or both in the network (Fig. S6C).

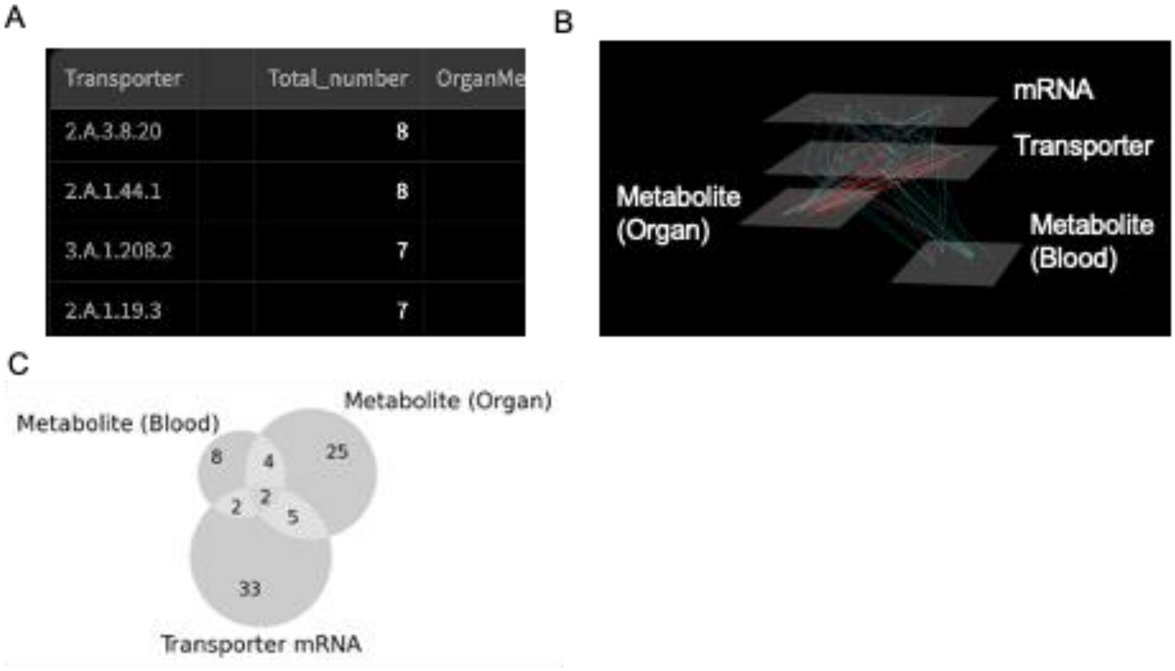

Figure S6. Outputs of "(D) Metabolite exchange network ".
(A) A table of differentially regulated transporters and associated differentially expressed molecules. Transporters are assumed to be affected by the amount of metabolites and regulation by changing the amount of transporter gene expression. This inference draws from analysis of data from The Transporter Classification Database (TCDB) (https://www.tcdb.org/). Each transporter is listed in descending order of degree in the network.
(B) The metabolite exchange network for differentially regulated transporters. Nodes and edges indicate differentially expressed molecules and differential regulated transporters, respectively. iTraNet also outputs the interactive network that allows users to move molecules contained in mRNA, Transporter, Metabolite (Organ), and Metabolite (Blood) layers.
(C) Venn diagram of the differentially regulated transporters. The number in each circle represents the number of differentially regulated transporters.

3. Comparison of iTraNet with other web-based tools

Multi-omics integration methods are categorized into two types: knowledge-driven and data-driven (Ewald *et al.*, 2024). Knowledge-driven methods analyze each omics layer individually, identifying molecular features that are mapped to knowledge bases. Data-driven approaches identify common patterns across omics layers, facilitating the discovery of new

relationships. iTraNet uses knowledge-driven methods to integrate multi-omics data, which can complement tools that use data-driven approaches, such as OmicsAnalyst (Zhou *et al.*, 2021).

Table S1 compares iTraNet with other tools. As previously described, various web applications have been developed to visualize and analyze multi-omics data (Zhou *et al.*, 2022, 2021). iTraNet complements these tools by incorporating comprehensive molecular interactions, including allosteric regulation and transporters. Metabolism is regulated by molecular interactions between cells via transporters and intracellular metabolism influenced by allosteric regulations. However, no other knowledge-driven tool incorporates allosteric regulations into the metabolic network, probably due to the cumbersome integration of the KEGG and BRENDA databases, which contain the allosteric regulatory network. iTraNet successfully incorporates allosteric regulations into the metabolic network and helps to understand metabolism by providing a more comprehensive analysis of cell-to-cell and intracellular regulatory relationships.

In addition, iTraNet excels in 2D/3D network visualization and analysis, effectively arranging multi-omics molecules within metabolic pathways, such as glycolysis/gluconeogenesis and the TCA cycle, using the well-known KEGG layout (Fig. S7). This feature is expected to facilitate a more intuitive understanding of intricate biological networks by researchers. While Arena3D (Kokoli *et al.*, 2023) lacks the ability to construct trans-omics networks and map them to KEGG pathway layouts, it can visualize 3D networks well. As shown in Figure S8, the output generated by iTraNet has some similarities to the input format required by Arena3D. We expect that this compatibility will provide an opportunity for researchers to use both tools synergistically. By modifying iTraNet’s output to serve as input for Arena3D, we can view the trans-omics network from different angles, providing a more comprehensive understanding of the network structure and interactions.

This combinatorial approach leverages the strengths of both tools, allowing researchers to explore the complex relationships within trans-omics data in a visually intuitive manner.

		iTraNet	OmicsNet	PaintOmics	OmicsAnalyst	Arena3D
Network creation		Knowledge-driven	Knowledge-driven	Knowledge-driven	Data-driven	User-defined
Estimated regulation	TF-mRNA	+	+	+	Correlation	-
	miRNA-mRNA	+	+	+	Correlation	-
	Protein-protein	+	+	+	Correlation	-
	Metabolite-enzyme	+	+	+	Correlation	-
	Allosteric regulation	+	-	-	Correlation	-
	mRNA-Transporter	+	-	-	Correlation	-
	Metabolite-transporter	+	-	-	Correlation	-
Network visualization	3D view	+	+	-	+	+
	KEGG-layout	+	-	+	-	-
Network analysis		+	+	+	+	+

Table S1. Comparison of iTraNet with other web-based tools for multi-omics integration. “+”, present; “-”, absent.

Collectively, given the growing interest in user-friendly multi-omics analysis tools (Kokoli *et al.*, 2023; Ewald *et al.*, 2024), we anticipate that the following four aspects (A-D) will demonstrate the novel nature of iTraNet and its significant impact on various fields of researchers.

A, Comprehensive integration of multi-omics data:

By incorporating gene regulatory networks, protein-protein interactions, metabolic networks, and metabolite exchange networks, iTraNet provides a more holistic view of cellular processes than existing tools. Of particular note is the inclusion of both allosteric regulation and transporter-mediated interactions in the metabolic modelling component. This approach can have a significant impact in capturing the complexity of metabolic regulation, as both of these mechanisms exert considerable influence on metabolite concentrations. We anticipate that iTraNet will be a valuable tool especially for researchers investigating metabolic disorders, cancer metabolism, and other pathological conditions characterized by metabolic dysregulation.

B, Intuitive visualization with KEGG layout:

iTraNet visualizes trans-omics networks using the KEGG layout, improving the interpretability of complex biological networks. We expect this feature to bridge traditional pathway analysis and network-based approaches, providing a more intuitive understanding of the data.

C, Accessibility and ease of use:

As a web-based platform, iTraNet makes complex trans-omics analysis accessible to a broader range of researchers, including those who may not have extensive computational expertise. This accessibility can significantly accelerate discoveries across various fields of biology and medicine.

D. Extensibility:

iTraNet's open-source and modular design allows for future extensions and enhancements. Researchers can modify and extend the platform, ensuring that it evolves with the field by incorporating new omics data and analysis methods. The code is available at https://github.com/HikaruSugimoto/Transomics_iTraNet.

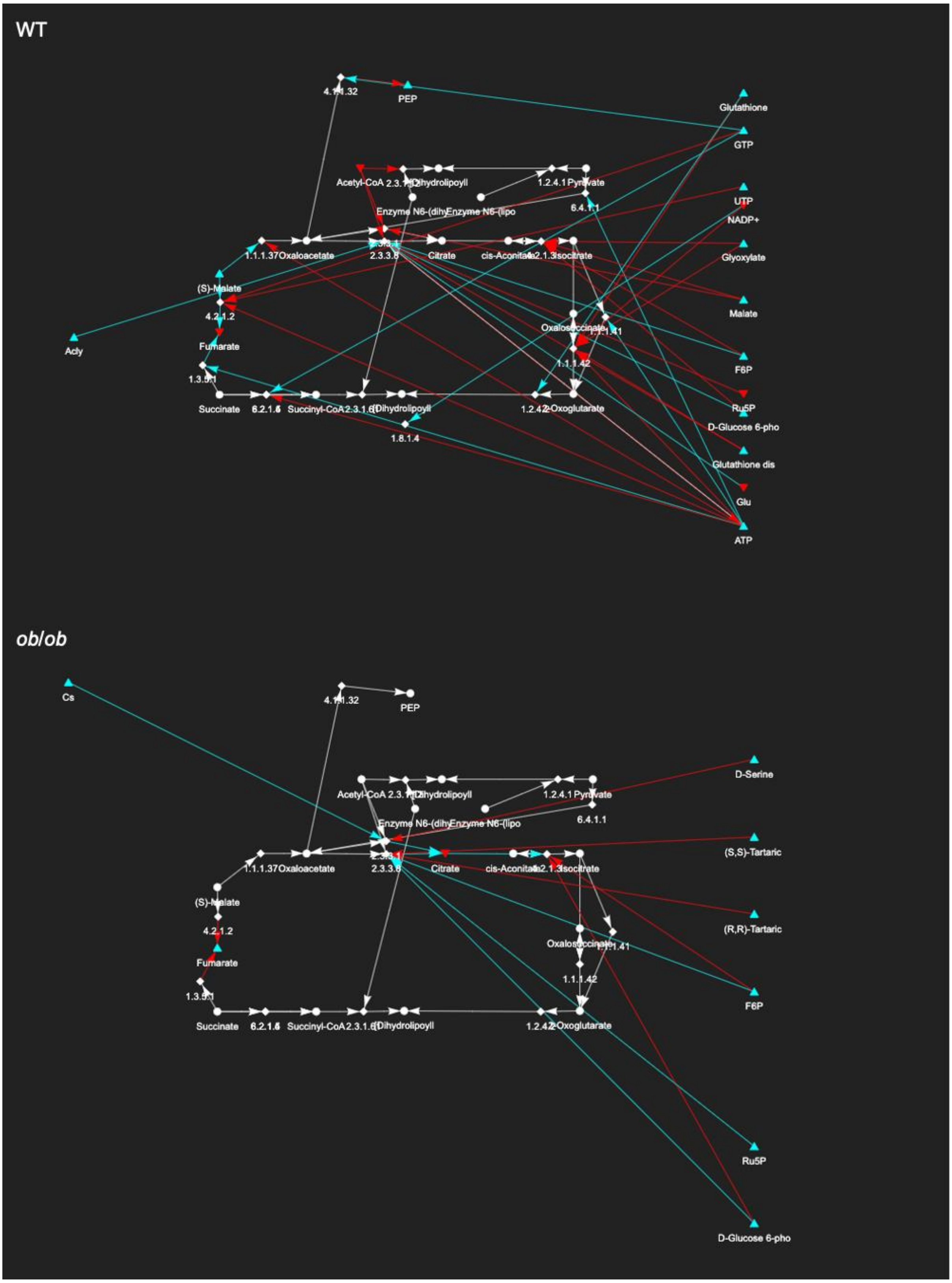
WT
4.1.1.32
PEP
Glutathione
GTP
Acetyl-CoA
1.2.4.1
Pyruvate
UTP
6.4.1.1
NADP+
1.1.1.37
Oxaloacetate
2.3.3.8
Citrate
cis-Aconitate
Isocitrate
Glyoxylate
(S)-Malate
4.2.1.2
Malate
Acly
Fumarate
Oxalosuccinate
1.1.1.41
F6P
1.1.1.42
1.3.5.1
Succinate
Succinyl-CoA
2-Oxoglutarate
Ru5P
D-Glucose 6-pho
1.8.1.4
Glutathione dis
Glu
ATP
ob/ob
Cs
4.1.1.32
PEP
D-Serine
Acetyl-CoA
1.2.4.1
Pyruvate
6.4.1.1
(S,S)-Tartaric
1.1.1.37
Oxaloacetate
2.3.3.8
Citrate
cis-Aconitate
Isocitrate
(S)-Malate
(R,R)-Tartaric
4.2.1.2
Oxalosuccinate
1.1.1.41
Fumarate
1.1.1.42
F6P
1.3.5.1
Succinate
Succinyl-CoA
2-Oxoglutarate
Ru5P
D-Glucose 6-pho

Figure S7. Metabolic networks.

Metabolic networks for differentially regulated metabolic reactions of WT and *ob/ob* mice in the TCA cycle. Nodes and edges indicate differentially expressed molecules and differentially regulated reactions, respectively. The differential regulations are classified into either activating (edges with "increased color" (cyan)) or inhibiting (edges with "decreased color" (red)). Arrows from metabolites in the right column to metabolic enzymes represent allosteric regulation, while arrows from mRNAs in the left column to metabolic enzymes indicate regulation by changes in mRNA levels.

Output of iTraNet

TF/miRNA	mRNA	FC	Regulation
Ep300	Sspn	UP	TF
Ep300	Stat3	UP	TF
Ep300	Rps29	UP	TF
Ep300	Ciart	UP	TF
Ep300	Pemt	UP	TF
Ep300	Coq10b	UP	TF

Input of Arena3D

SourceNode	TargetNode	Weight	SourceLayer	TargetLayer
2	1	1	Layer1	Layer1
3	2	1	Layer1	Layer1
4	2	1	Layer1	Layer1
5	4	1	Layer1	Layer1
6	2	1	Layer1	Layer1
7	3	1	Layer1	Layer1

Figure S8. Comparison between iTraNet output and Arena3D input formats.

The iTraNet output provides information on network connectivity, specifying the directionality of connections between nodes. It also categorizes each node by molecule type, such as transcription factor (TF), miRNA, or mRNA, as shown in this example. The output also includes data on the up- or down-regulation status of these molecules. This information set includes all the necessary elements required for input to Arena3D, facilitating seamless integration between the two platforms.

Supplementary References

Chang,A. *et al.* (2021) BRENDA, the ELIXIR core data resource in 2021: new developments and updates. *Nucleic Acids Res.*, **49**, D498–D508.

Egami,R. *et al.* (2021) Trans-omic analysis reveals obesity-associated dysregulation of inter-organ metabolic cycles between the liver and skeletal muscle. *iScience*, **24**, 102217.

Ewald,J.D. *et al.* (2024) Web-based multi-omics integration using the Analyst software suite. *Nat. Protoc.* 1–31.

Ge,S.X. *et al.* (2018) iDEP: an integrated web application for differential expression and pathway analysis of RNA-Seq data. *BMC Bioinformatics*, **19**, 534.

Goentoro,L. *et al.* (2009) The incoherent feedforward loop can provide fold-change detection in gene regulation. *Mol. Cell*, **36**, 894–899.

Huang,H.-Y. *et al.* (2021) miRTarBase update 2022: an informative resource for experimentally validated miRNA–target interactions. *Nucleic Acids Res.*, **50**, D222–D230.

Kanehisa,M. *et al.* (2017) KEGG: new perspectives on genomes, pathways, diseases and drugs. *Nucleic Acids Res.*, **45**, D353–D361.

Kawata,K. *et al.* (2018) Trans-omic Analysis Reveals Selective Responses to Induced and Basal Insulin across Signaling, Transcriptional, and Metabolic Networks. *iScience*, **7**, 212–229.

Kinsella,R.J. *et al.* (2011) Ensembl BioMarts: a hub for data retrieval across taxonomic space. *Database* , **2011**, bar030.

Kokaji,T. *et al.* (2022) In vivo transomic analyses of glucose-responsive metabolism in skeletal muscle reveal core differences between the healthy and obese states. *Sci. Rep.*, **12**, 13719.

Kokaji,T. *et al.* (2020) Transomics analysis reveals allosteric and gene regulation axes for altered hepatic glucose-responsive metabolism in obesity. *Sci. Signal.*, **13**.

Pang,Z. *et al.* (2022) Using MetaboAnalyst 5.0 for LC–HRMS spectra processing, multi-omics integration and covariate adjustment of global metabolomics data. *Nat. Protoc.*, **17**, 1735–1761.

Saier,M.H. *et al.* (2021) The Transporter Classification Database (TCDB): 2021 update. *Nucleic Acids Res.*, **49**, D461–D467.

Szklarczyk,D. *et al.* (2022) The STRING database in 2023: protein–protein association networks and functional enrichment analyses for any sequenced genome of interest. *Nucleic Acids Res.*, **51**, D638–D646.

Terakawa,A. *et al.* (2022) Trans-omics analysis of insulin action reveals a cell growth sub-network which co-regulates anabolic processes. *iScience*. **25**.

Yugi,K. *et al.* (2014) Reconstruction of insulin signal flow from phosphoproteome and metabolome data. *Cell Rep.*, **8**, 1171–1183.

Yugi,K. *et al.* (2016) Trans-omics: how to reconstruct biochemical networks across multiple ‘omic’layers. *Trends Biotechnol.*, **34**, 276–290.

Zhou,G. *et al.* (2021) OmicsAnalyst: a comprehensive web-based platform for visual analytics of multi-omics data. *Nucleic Acids Res.*, **49**, W476–W482.

Zhou,G. *et al.* (2022) OmicsNet 2.0: a web-based platform for multi-omics integration and network visual analytics. *Nucleic Acids Res.*, **50**, W527-33.

Zou,Z. *et al.* (2022) ChIP-Atlas 2021 update: a data-mining suite for exploring epigenomic landscapes by fully integrating ChIP-seq, ATAC-seq and Bisulfite-seq data. *Nucleic Acids Res.*, **50**, W175–W182.
